# *MicroRNA‐183‐5p* is stress‐inducible and protects neurons against cell death in amyotrophic lateral sclerosis

**DOI:** 10.1111/jcmm.15490

**Published:** 2020-06-18

**Authors:** Chunyu Li, Yongping Chen, Xueping Chen, Qianqian Wei, Ruwei Ou, Xiaojing Gu, Bei Cao, Huifang Shang

**Affiliations:** ^1^ Department of Neurology National Clinical Research Center for Geriatrics West China Hospital Sichuan University Chengdu China

**Keywords:** amyotrophic lateral sclerosis, cell death, cell stress, *miR‐183‐5p*

## Abstract

Amyotrophic lateral sclerosis (ALS) is a fatal neurodegenerative disease characterized by the death of motor neurons. A fundamental pathogenesis of ALS is the prolonged cell stress in neurons, which is caused by either accumulation of protein aggregates or reactive oxygen species. However, the mechanistic link between stress sensing and cell death is unsettled. Here, we identify that *miR‐183‐5p*, a neuron‐enriched miRNA, couples stress sensing and cell death programming in ALS. *miR‐183‐5p* is immediately induced by hydrogen peroxide, tunicamycin or TNF‐α in neurons. The overexpression of *miR‐183‐5p* increases neuron survival under stress conditions, whereas its knockdown causes neuron death. *miR‐183‐5p* coordinates apoptosis and necroptosis pathways by directly targeting *PDCD4* and *RIPK3*, and thus protects neurons against cell death under stress conditions. The consistent reduction of *miR‐183‐5p* in ALS patients and mouse models enhances the notion that *miR‐183‐5p* is a central regulator of motor neuron survival under stress conditions. Our study supplements current understanding of the mechanistic link between cell stress and death/survival, and provides novel targets for clinical interventions of ALS.

## 1 INTRODUCTION

Amyotrophic lateral sclerosis (ALS) is a fatal motor neuron disease characterized by the progressive degeneration of motor neurons in the brain and spinal cord.^1^, ^2^ A pathological hallmark of motor neuron degeneration in ALS is the accumulation of aggregated proteins and concomitant endoplasmic reticulum stress (ER stress) and oxidative stress.^3^, ^4^ Assessment of distinct cell stress levels is potential to be biomarkers for the diagnosis of ALS.^5^, ^6^ Beyond diagnostic application, mechanistic studies on stress response in ALS also provide therapeutic targets.^3^, ^7^, ^8^ Thus, investigations into the diverse mechanisms of neuronal response to cellular stress are beneficial to identify therapeutic targets for ALS.

In the central nervous system, neuron is highly activated and faces many energetic challenges. Therefore, neuron is frequently exposed to stressful environments and particularly susceptible to stress‐induced damages.^9^, ^10^ Neuronal intrinsic and environment‐induced cellular stress causes a broad spectrum of structural and functional changes, such as protein aggregation, mitochondrial dysfunction and programmed cell death, which are shared in neurodegenerative diseases including ALS.^11^, ^12^ Recent discoveries shed light on critical functions of stress response in neurodegeneration, with an emerging realization that failure of stress response in neurons influences the initiation and progression of neurodegenerations disorders.^13^, ^14^

Neuronal stress response relies on a complicated network of stress response pathways, including autophagy, unfolded protein response and multiple signalling pathways.^14^ Emerging evidence establishes that perturbations in non‐coding RNAs (ncRNAs) are involved in stress sensing and neuron death/survival. In particular, microRNAs (miRNAs) that regulate gene expression either by translational inhibition or targeted mRNA cleavage^15^, ^16^ are extensively altered in neurodegenerative diseases.^17^, ^18^ The expression of miRNA is dynamically regulated, and thus miRNA functions in stress responses.^19^ Therefore, miRNA is positioned at the coupling of cell stress sensing and response. *miR‐183‐5p*, together with *miR‐96‐5p* and *miR‐182‐5p*, comprises the *miR‐183/96/182* cluster, which is expressed in neural cells and involved in long‐term memory^20^ and neuropathic pain.^21^ Here, we report that *miR‐183‐5p* is highly enriched in motor neurons and coordinates stress sensing and cell death in ALS.

## 2 MATERIALS AND METHODS

### 2.1 Cell culture

Cortical neuron cultures were based on the original methods described by Kaech and Bankerde.^22^ In brief, cerebral cortex from mouse embryos of 17.5 days was dissected and digested with Papain (Worthington) and DNAse (Sigma) at 37°C for 15 minutes. The dissociated neurons were plated in 6‐well culture plates with a density of 1 × 10^6^ cells per well for further experiments.^23^ Astroglia were cultured from cerebral cortex from newborn mouse as described.^22^ Briefly, cerebral cortex was dissected free of meninges and digested with 0.25% trypsin (Gibco) at 37°C. Then, the tissue pieces were dissociated and planted in 75 cm^2^ in MEM (Gibco) supplemented with 10% Horse Serum (Hyclone), 1% Glutamine (Invitrogen) and antibiotics (Gibco). The astroglia cultures were fed every 3 days harvested at the 80% confluence.

Neuroblastoma cell line Neuro2a was purchased from American Type Culture Collection (ATCC), and NSC‐34 was provided by Dr NR Cashman (University of Toronto, Toronto, Canada).^24^ These cells were maintained by DMEM (Gibco) plus 10% FBS and antibiotics. For in vitro experiments, cells treated with H_2_O_2_ 200 μmol/L for 2 hours or tunicamycin (Sigma) 1 μg/mL for 2 hours or mouse TNF‐α 100 ng/mL for 24 hours, respectively, and further detections were conducted. For the transfections, NSC‐34 cells were transfected with *miRNA* mimics, inhibitors or scramble sequences by RNAiMAX following instructions.

### 2.2 RNA extraction and RT‐qPCR assay

Total RNAs, including miRNA, were extracted and collected using TRIzol. The RNA quality and quantity were measured by NanoDrop 2000c (Thermo). The reverse‐transcription PCR and qPCR for miRNA were performed following standard protocols as described previously.^25^ The expression of miRNA was normalized to U6, quantified by the 2^−ΔΔCt^ method.

### 2.3 *Fluorescence* in situ* hybridization*

Fluorescence in situ hybridization was carried out as previously described.^26^ Briefly, the frozen tissue sections were thawed, then digested with pepsin and hybridized with a biotin‐labelled probe corresponding to mature *miR‐183‐5p* or negative control. Finally, DAPI was used to stain the nuclei. Images were acquired using a confocal laser‐scanning microscope (ZEISS 880+ Airyscan).

### 2.4 Bioinformatics analysis

The target gene of *miR‐183‐5p* was predicted with four different online databases, including microRNA.org (http://www.microrna.org/microrna/microrna),^27^ Targetscan (http://www.targetscan.org/vert_71/),^28^ Diana Tools (http://diana.imis.athena‐innovation.gr/DianaTools/index.php)^29^ and miRDB (http://www.mirdb.org/miRDB/).^30^ The target genes predicted were used for the GO (Gene Ontology) term analysis in Gene Ontology Consortium (http://geneontology.org/) and DAVID Bioinformatics Resources (https://david.ncifcrf.gov/).

### 2.5 Cell death assay

The cell death was examined by flow cytometry using Annexin V‐APC/7‐AAD kit (4A Biotech) according to the manufacturer's protocol. In brief, NSC‐34 cells were transfected with *miR‐183‐5p* inhibitors, mimics or scramble sequence (100 nmol/L) for 24 hours and then treated with H_2_O_2_ 200 μmol/L for 2 hours or tunicamycin 1 μg/mL for 2 hours or TNF‐α 100 ng/mL for 24 hours, respectively. The NSC‐34 cells were stained for 20 minutes in dark and then analysed by flow cytometry.

### 2.6 Dual‐luciferase reporter assay

To examine the interaction between *miR‐183‐5p* and *PDCD4* or *RIPK3*, plasmids of wild type or mutant sequence of 3′UTR of *PDCD4* or *RIPK3* and *miR‐183‐5p* mimics or negative control (miR‐NC) were cotransfected to NSC‐34 cells by Lipofectamine 2000 (Thermo). 48 hours after transfection, the cells were harvested and the luciferase activities were measured. The relative Renilla luciferase activity was normalized to firefly luciferase activity.

### 2.7 Protein extraction and western blots

Protein extraction and Western blots were performed as previously reported.^25^ In brief, equivalent proteins in each group were isolated using SDS‐PAGE and transferred to PVDF membranes (Millipore). Subsequently, the membranes were blocked with 5% skim milk on a rocker and then incubated with primary antibodies at 4°C overnight, followed by appropriate second antibody (Abcam) for 2 hours. Finally, the membranes were treated with ECL reagent (Thermo Fisher Scientific) for exposure. The ImageJ software was used for optical densities quantification. The primary antibody included anti‐PDCD4 (Cell Signalling Technology, #9535), anti‐RIPK3 (Cell Signalling Technology, #95702), anti‐Cleaved Caspase 3 (Abcam, ab214430), anti‐p‐MLKL (Abcam, ab196436) and anti‐β‐Actin (Abcam, ab179467).

### 2.8 SOD1^G93A^ transgenic mice

SOD1^G93A^ transgenic mice were purchased from the Jackson Laboratory. Mice were monitored weekly and staged according to previously report.^31^ The pre‐symptomatic stage (Pre‐sym), early‐symptomatic stage (Early‐sym) and late‐symptomatic (Late‐sym) *SOD1* ^G93A^ mice were used for experiments and gender‐matched littermates were used as controls. Mouse experiments were conducted in compliance with the Laboratory Animal Care Guidelines authorized by Sichuan University.

### 2.9 Statistical analysis

GraphPad Prism 7.0 software was used for statistical analysis. All quantitative data were shown as the mean ± SEM unless otherwise stated. Significant statistical differences between the two groups were determined by the two‐tailed unpaired Student's *t* test and for three or more groups, one‐way ANOVA (followed by a Dunnett post hoc test) was used for statistical analysis. The *P*‐value < .05 was assumed as statistically significant.

## 3 RESULTS

### 3.1 *miR‐183‐5p* is enriched in neurons of the spinal cord and dynamically regulated in ALS mice

**Figure 1:**
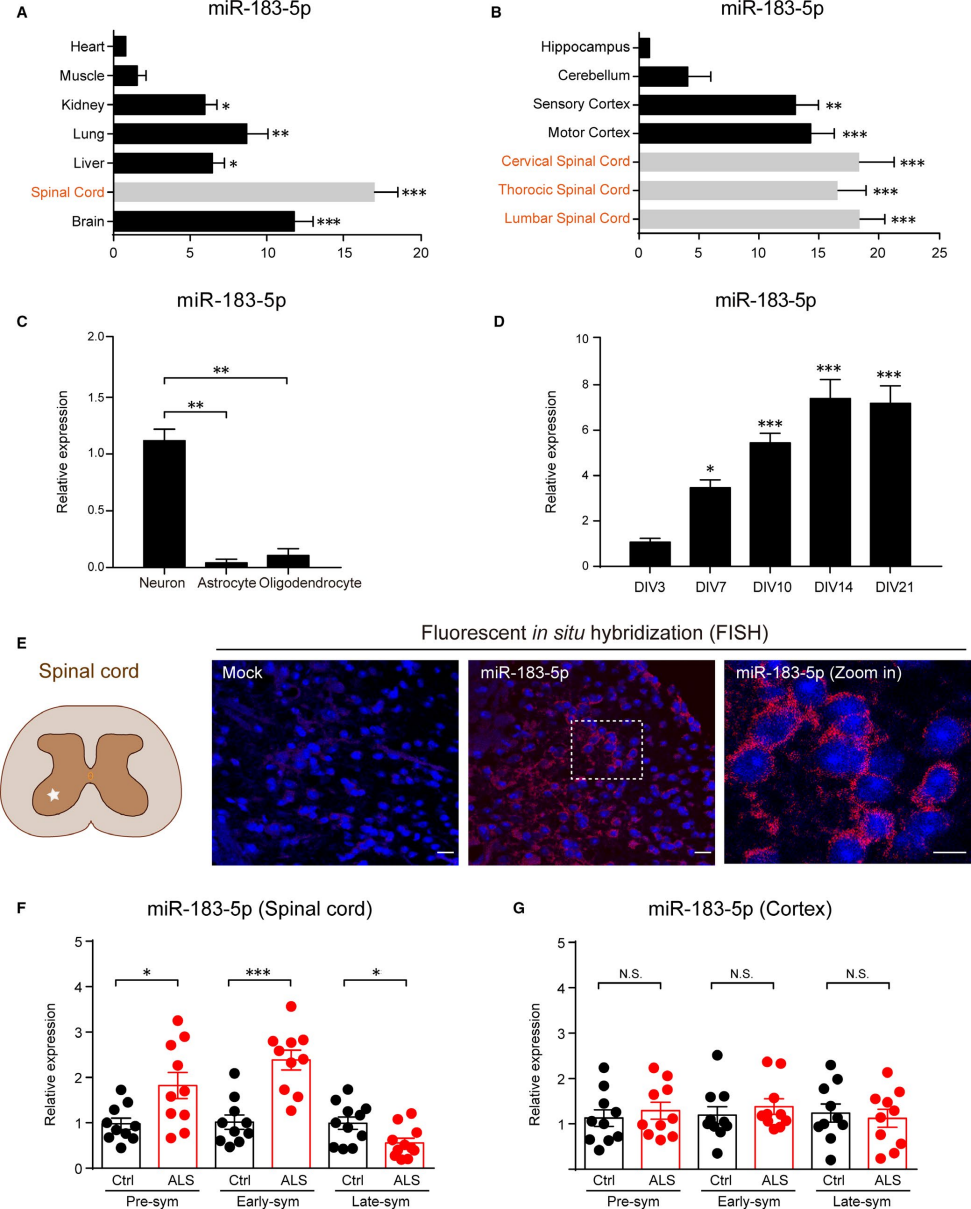
*miR‐183‐5p* is enriched in neurons and dynamically altered in progression of ALS mice. A and B, RT‐qPCR shows the expression pattern of *miR‐183‐5p* in multiple organs (A) and different part of central nervous system (B). Data represent mean ± SEM. *P < .05, **P < .01 and ***P < .001, n = 6. C, RT‐qPCR result indicates the expression of *miR‐183‐5p* in neurons, astrocytes and oligodendrocytes. Data represent mean ± SEM. *P < .05, **P < .01 and ***P < .001, n = 3. D, RT‐qPCR shows the *miR‐183‐5p* expression in the primary neurons during the formation of neuronal network. Data represent mean ± SEM. *P < .05, **P < .01 and ***P < .001, n = 3. E, Fluorescent in situ hybridization (FISH) analysis of *miR‐183‐5p* expression in the grey matter of the anterior horn of spinal cord. Scale bar 25 μm. F and G, RT‐qPCR results shows the dynamic changes of miR‐183‐5p in the lumbar spinal cord (F) and motor cortex (G). Pre‐sym: pre‐symptomatic stage, Early‐sym: early‐symptomatic stage, Late‐sym: late‐symptomatic stage. Data represent mean ± SEM. *P < .05, **P < .01 and ***P < .001. n = 10

As the first step towards understanding the role of *miR‐183‐5p* in neural systems, we performed reverse transcription quantitative PCR (RT‐qPCR) to investigate *miR‐183‐5p* expression pattern in different organs of mouse. Results showed that *miR‐183‐5p* was generally expressed in multiple organs/tissues, particularly in neural tissues, such as spinal cord and brain (Figure 1A). It's noted that *miR‐183‐5p* was extremely enriched in the spinal cord. Thus, we studied the neural expression of *miR‐183‐5p* and found that *miR‐183‐5p* was highly expressed in the lumbar, thoracic and cervical spinal cord, but lower in the hippocampus and cerebellum (Figure 1B). By primary cultures of neurons, astrocytes and oligodendrocytes, we found that neuronal expression of *miR‐183‐5p* was much higher than its glial counterparts (Figure 1C). The expression of *miR‐183‐5p* was increased during the formation of neuronal network (Figure 1D). The neural expression of *miR‐183‐5p* was recapitulated by fluorescent in situ hybridization (FISH), showing that *miR‐183‐5p* was highly expressed in the anterior horn of spinal cord, where motor neuron resides (Figure 1E).

The expression of *miR‐183‐5p* in the spinal cord indicates its implication in motor neuron disease, including ALS. To test this hypothesis, we examined its expression pattern in animal models of ALS. Results showed that *miR‐183‐5p* was increased in the spinal cord of ALS mice in the pre‐symptomatic and early symptomatic stages and reciprocally decreased in the late symptomatic stage of disease (Figure 1F). However, the expression of *miR‐183‐5p* was not dramatically altered in the mouse cortex (Figure 1G). All data reveal the neuronal enrichment of *miR‐183‐5p* in the spinal cord, implying its potential role in ALS.

### 3.2 *miR‐183‐5p* expression is dynamically regulated by cell stress

**Figure 2:**
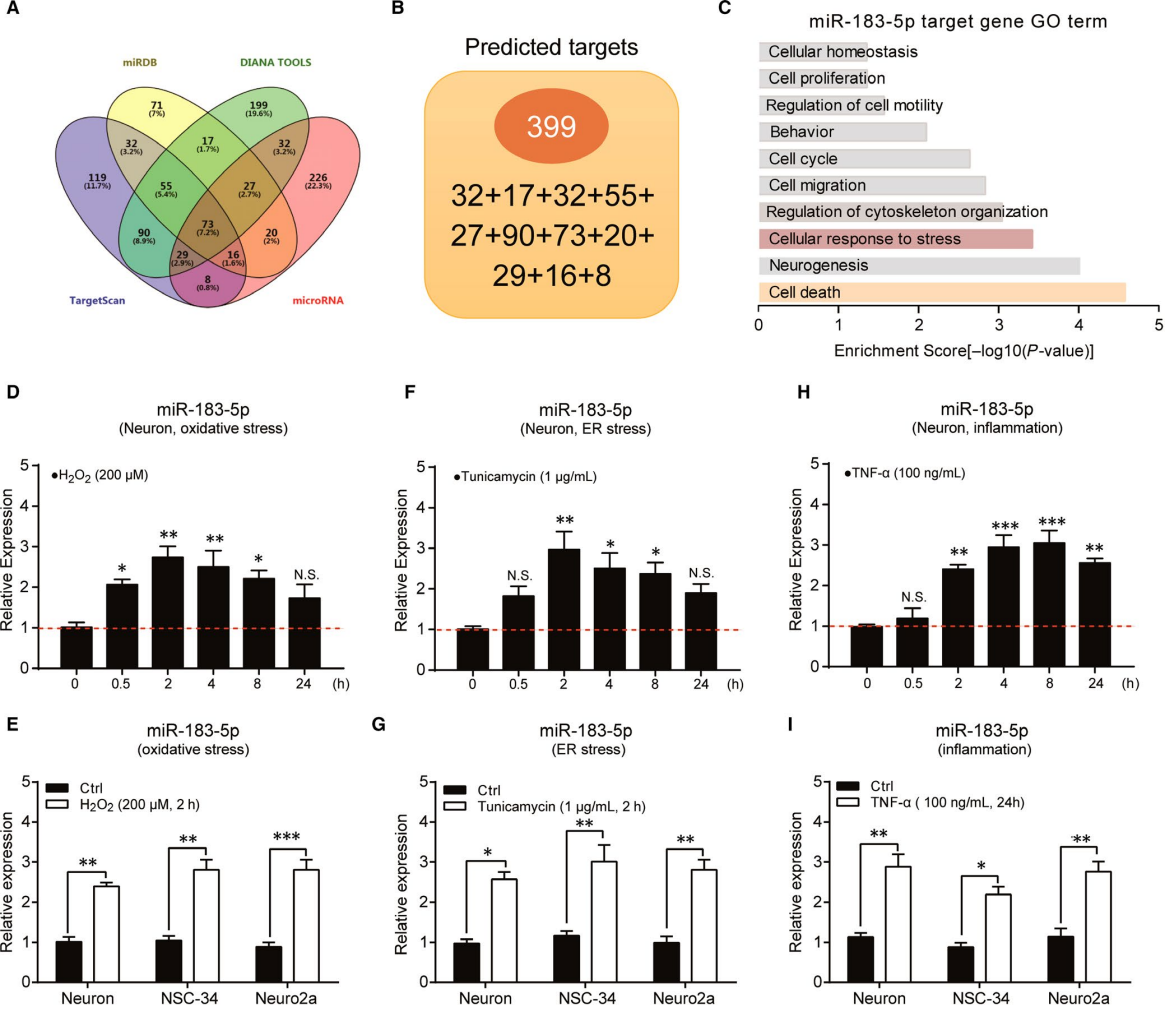
*miR‐183‐5p* expression is dynamically regulated by cell stress. A and B, Overlap analysis of *miR‐183‐5p* targets predicted by four different miRNA prediction tools with VENNY (A) and totally 399 targets predicted by at least two different databases (B). C, Go term analysis of the *miR‐183‐5p* target genes. D and E, *miR‐183‐5p* is induced by oxidative stress evoked by H_2_O_2_ in primary neurons, NSC‐34 cells and Neuro2a cells. F and G, *miR‐183‐5p* is induced by ER stress aroused by tunicamycin in primary neurons, NSC‐34 cells and Neuro2a cells. H and I, *miR‐183‐5p* is induced by inflammatory stress mimicked by TNF‐α treatment. Data represent mean ± SEM. *P < .05, **P < .01 and ***P < .001. n = 3

To better understand the function of *miR‐183‐5p* in neurons, we performed bioinformatic prediction to screen the downstream genes targeted by *miR‐183‐5p*. By screening databases of microRNA.org,^27^ Targetscan,^28^ Diana Tools^29^ and miRDB,^30^ we found a total of 399 potential genes targeted by *miR‐183‐5p* in at least two databases, which were concentrated in the pathways of cell stress responses and cell death by GO (Gene Ontology) analysis (Figure 2A‐C). Thus, we next examined the expression of *miR‐183‐5p* in response to cellular stress. Neuronal *miR‐183‐5p* was induced by H_2_O_2_ that evokes oxidative stress, and this induction was recapitulated in NSC‐34 and Neuro2a cells (Figure 2D,E). It's also noticed that the inductive *miR‐183‐5p* by oxidative stress was quickly increased within 30 minutes after H_2_O_2_ treatment. (Figure 2D).

To further investigate the dynamic regulation of *miR‐183‐5p*, we next examined its response to ER stress. Results showed that neuronal *miR‐183‐5p* was increased in close temporal association with ER stress occurring by tunicamycin (TM) (Figure 2F). Similarly, the inductive *miR‐183‐5p* to ER stress in neurons was reproduced in NSC‐34 and Neuro2a cells (Figure 2G). Neural inflammation activates cell stress and causes neuronal death and degeneration. We wondered whether *miR‐183‐5p* was dynamically controlled by external inflammatory cytokines. By TNF‐α treatment, we found that *miR‐183‐5p* was increased in neuron cultures as well as NSC‐34 and Neuro2a cells (Figure 2H,I). Taken together, our data indicate the dynamic regulation of *miR‐183‐5p* by cell stress in neurons.

### 3.3 *miR‐183‐5p* protects against cell death under stress conditions

**Figure 3:**
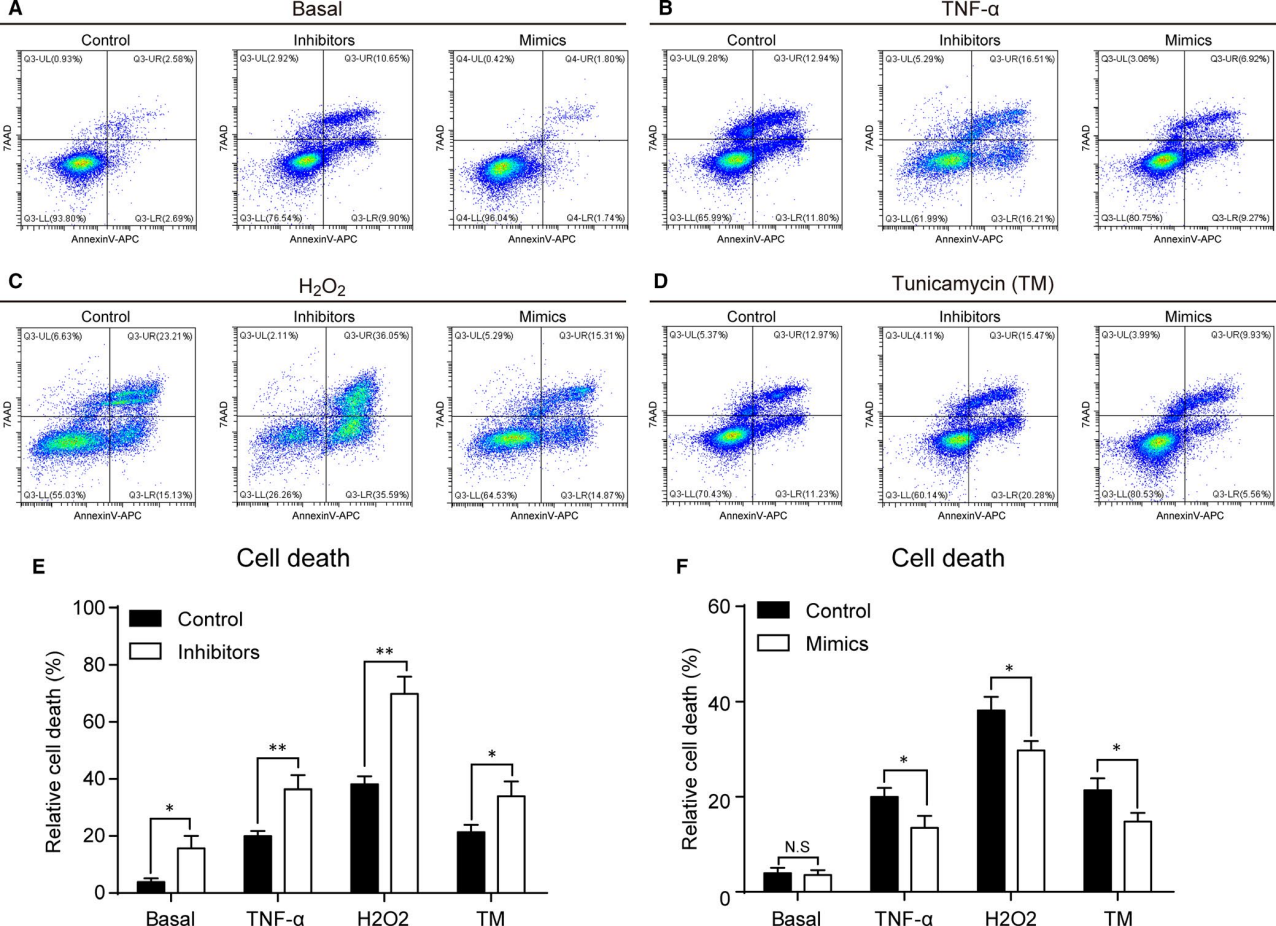
*miR‐183‐5p* desensitizes cell to death under stress conditions. A‐D, Flow cytometry assay shows that *miR‐183‐5p* mimics protect cells from cell death while inhibitors induce cell death at basal (A) and TNF‐α (B), H_2_O_2_ (C) and tunicamycin (TM) (D) treatment conditions. E and F, The total cell death is statistically analysed in inhibitors (E) and mimics (F) respectively. Data represent mean ± SEM. *P < .05, **P < .01 and ***P < .001, n = 3

Considering the neuronal enrichment and stress‐responsive regulation of *miR‐183‐5p*, we hypothesized that *miR‐183‐5p* might play a role in protecting neurons against cell stress. We knocked down or overexpressed *miR‐183‐5p* by transfection of its mimics or inhibitors in NSC‐34 cells (Figure S1). The cell death was examined by flow cytometry assay. Under basal conditions, *miR‐183‐5p* knockdown slightly increased percentages of cell death in NSC‐34 cells (Figure 3A,E,F). However, *miR‐183‐5p* knockdown dramatically increased cell death in NSC‐34 cells treated by TNF‐α, H_2_O_2_ and tunicamycin, whereas *miR‐183‐5p* overexpression promoted cell survival under stress conditions (Figure 3B‐F). Therefore, *miR‐183‐5p* is indispensable for cell survival under stress conditions in neuronal cells.

### 3.4 *miR‐183‐5p* targets *RIPK3* and *PDCD4* to regulate cell death

**Figure 4:**
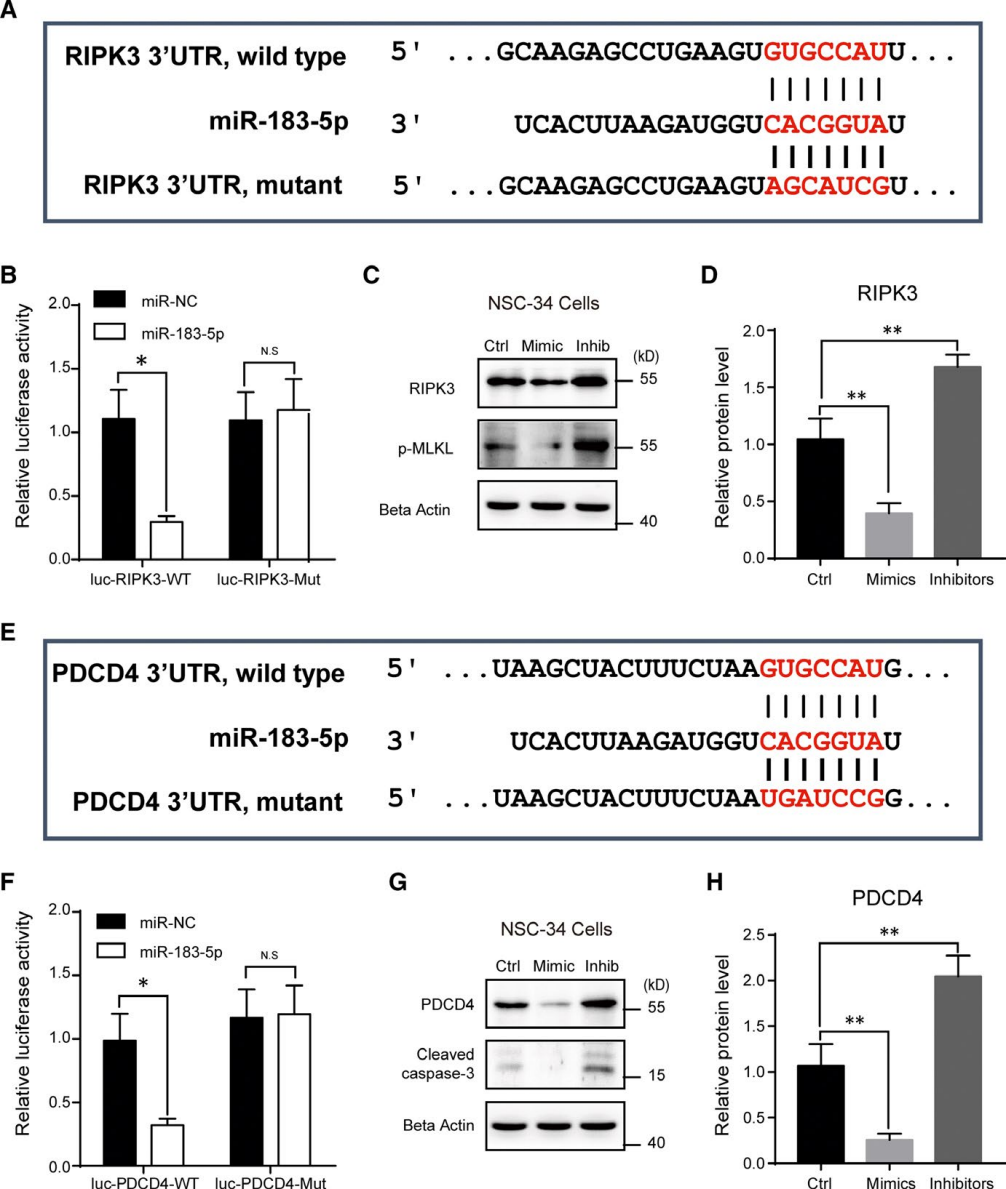
*miR‐183‐5p* targets *RIPK3* and *PDCD4*. A, Sequence analysis of *miR‐183‐5p* binding with the 3′‐UTR of *PDCD4*. B, Luciferase report assay showing the reduction of luciferase activity in NSC‐34 cells with wild‐type *PDCD4*, but not the mutant. C and D, Western blots showing the protein levels of *PDCD4* in NSC‐34 cells transfected with *miR‐183‐5p* mimics, inhibitors or scramble sequence (100 nmol/L, respectively). E, Sequence analysis of *miR‐183‐5p* binding with the 3′‐UTR of *RIPK3*. F, Luciferase report assay showing the reduction of luciferase activity in NSC‐34 cells with wild‐type *RIPK3*, but not the mutant. G and H, Western blots showing the protein levels of *RIPK3* in NSC‐34 cells transfected with *miR‐183‐5p* mimics, inhibitors or scramble sequence (100 nmol/L, respectively). Results were averages of four independent experiments. Data represent mean ± SEM. *P < .05, **P < .01 and ***P < .001, n = 3

To clarify how *miR‐183‐5p* participates in regulation of neuronal survival/death, we next examined the potential targets of *miR‐183‐5p*. By sequence analysis, we found that *RIPK3*, a well‐known regulator of necroptosis, was a potential candidate of *miR‐183‐5p* in NSC‐34 cells (Figure 4A). Results of luciferase reporter assay confirmed the binding of *miR‐183‐5p* with *RIPK3* (Figure 4B). To further validate the regulatory effect of *miR‐183‐5p* on *RIPK3*, we investigated *RIPK3* protein level in NSC‐34 cells. Western blots showed that the protein level of *RIPK3* was decreased by *miR‐183‐5p* overexpression and increased by its knockdown (Figure 4C,D). The consistent alternations of phospho‐MLKL confirmed the effect of *miR‐183‐5p* on *RIPK3*.

Moreover, we found that *miR‐183‐5p* also binds to *PDCD4*, a critical protein in cell apoptosis (Figure 4E). Similarly, luciferase reporter assay (Figure 4F) and Western blots (Figure 4G,H) demonstrated that *miR‐183‐5p* directly bound to *PDCD4* and negatively regulated the expression of PDCD4. Furthermore, the similar change patterns of cleaved caspase‐3 confirmed the effect of *miR‐183‐5p* on PDCD4. Therefore, we propose that *miR‐183‐5p* targets both *RIPK3* and *PDCD4* and coordinates necroptosis and apoptosis pathways to control neuronal survival/death.

## 4 DISCUSSION

**Figure 5:**
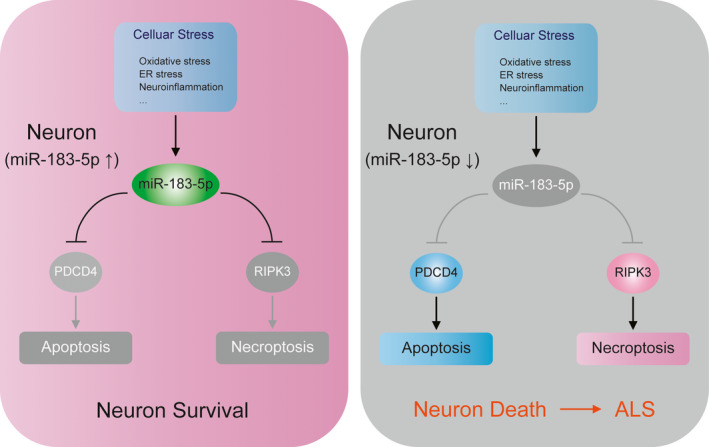
Model. A schematic model highlighting the role of *miR‐183‐5p* coupling the stress and cell death by directly targeted *PDCD4* and *RIPK3*

Emerging evidence establishes that neuronal stress responses are essential in neurodegenerative disease.^14^ The complicated relationship among stress sensing and responses, adaptive outcomes and neurodegeneration creates challenges for understanding the mechanistic interplay. In this study, we demonstrate that *miR‐183‐5p*, a neuron‐enriched miRNA, plays a vital role in the coupling of stress sensing and scavenging. *miR‐183‐5p* is highly expressed in neurons of the central nervous system and immediately induced in response to oxidative stress and ER stress. Elevation of *miR‐183‐5p* increases cell survival under stress conditions, whereas its knockdown promotes cell death. Mechanistically, *miR‐183‐5p* controls apoptotic and necroptotic pathways by directly targeting RIPK3 and PDCD4 and thus acts as a regulator of programmed neuron death (Figure 5). Our study identifies *miR‐183‐5p* as a nodal point of stress sensing and responding in motor neurons.

As a result of complicated architecture and energetic remodelling, neurons are susceptible to stress‐induced damages and thus develop distinctive responses to counteract cellular stress. An efficient coordination of neuronal stress sensing and pro‐survival signalling is critical for neuronal survival. There are many response pathways in neuronal stress responding, from gene expression to signalling cascade. Within these studies, miRNA has been widely recognized as a hallmark of neuronal stress response. Current evidence implicates that miRNAs play an important role in neurodegenerative diseases.^32^, ^33^, ^34^ Moreover, miRNAs are involved in pathways that regulate redox biology.^35^, ^36^ In this study, we propose that *miR‐183‐5p* is a protective factor in neuron survival. The fact that *miR‐183‐5p* is stress‐inducible indicates *miR‐183‐5p* is an immediately response factor to neuronal stress. Moreover, *miR‐183‐5p* suppresses neuronal death by controlling two distinct cell death pathways. Therefore, *miR‐183‐5p* coordinates stress sensing and responding in neurons and is critical for motor neuron survival under stress conditions.

So far, multiple gene mutations of ALS, such as *SOD1*, *TARDBP*, *FUS* and *TBK1*, have been identified.^37^ Regardless of how the primary gene defects are categorized, most of them are associated with common downstream pathologic processes, such as oxidative stress, ER stress and inflammatory stress, which finally cause neuronal death through apoptosis and necroptosis.^38^ However, the mechanistic link between the neuronal stress and death is not fully understood. Our results reveal that *miR‐183‐5p* is not only a stress sensor in motor neurons, but also an executive factor in neuron death programming. *miR‐183‐5p* modulates cell apoptosis by targeting *PDCD4* and necroptosis by *RIPK3*. In ALS, necroptosis is appreciated as a major pathway for motor neuron death.^39^ Therefore, *miR‐183‐5p* is critical for motor neuron survival and increased expression of *miR‐183‐5p* may be beneficial for motor neuron survival in ALS.

Our previous study investigated the miRNA expression profiles of Chinese ALS patients to explore novel biomarkers for ALS diagnosis. We found that *miR‐183‐5p* was down‐regulated in ALS patients and provided high diagnostic accuracy for ALS.^40^ In this study, we examined *miR‐183‐5p* expression pattern in the progression of ALS mouse. Intriguingly, we found that the increase of *miR‐183‐5p* is correlated with cell stress in motor neurons of ALS in pre‐symptomatic and early‐symptomatic stages, while in the late‐symptomatic stage *miR‐183‐5p* was decreased, possibly due to the progressive motor neuron degeneration/death.^41^ This finding is similar to our previous work, demonstrating the dynamic changes of a neuronal protective gene *LanCL1* in the progression of ALS.^23^ Therefore, the dynamic expression of *miR‐183‐5p* affords a novel diagnostic and therapeutic target for ALS.

In conclusion, we report that *miR‐183‐5p*, a neuronal enriched miRNA, is stress‐inducible and protects neurons against cell death. Our study supplements current understandings of the mechanistic link between cell stress and cell death/survival and provides novel targets for ALS interventions.

## CONFLICT OF INTEREST

The authors confirm that there are no conflicts of interest.

## AUTHOR CONTRIBUTION

**Chunyu Li:** Conceptualization (lead); Formal analysis (lead); Funding acquisition (equal); Investigation (lead); Methodology (lead); Supervision (equal); Writing‐original draft (lead); Writing‐review & editing (lead). **Yongping Chen:** Data curation (equal); Funding acquisition (supporting); Methodology (equal); Software (supporting). **Xueping Chen:** Data curation (equal); Formal analysis (equal); Resources (equal). **Qianqian Wei:** Resources (equal); Validation (equal); Visualization (equal). **Ruwei Ou:** Formal analysis (equal); Methodology (equal); Resources (equal). **Xiaojing Gu:** Data curation (equal); Methodology (equal). **Bei Cao:** Resources (equal); Validation (equal). **Huifang Shang:** Conceptualization (lead); Funding acquisition (equal); Project administration (lead); Supervision (lead); Writing‐review & editing (equal).

## Supporting information

Fig S1

## Data Availability

The data that support the findings of this study are available from the corresponding author upon reasonable request.
