# Supplementary figures and images for: MicroRNA‐183‐5p is stress‐inducible and protects neurons against cell death in amyotrophic lateral sclerosis
Source: J Cell Mol Med. 2020 Jun 18;24(15):8614–22. doi: 10.1111/jcmm.15490 (PMC7412410; doi:10.1111/jcmm.15490)

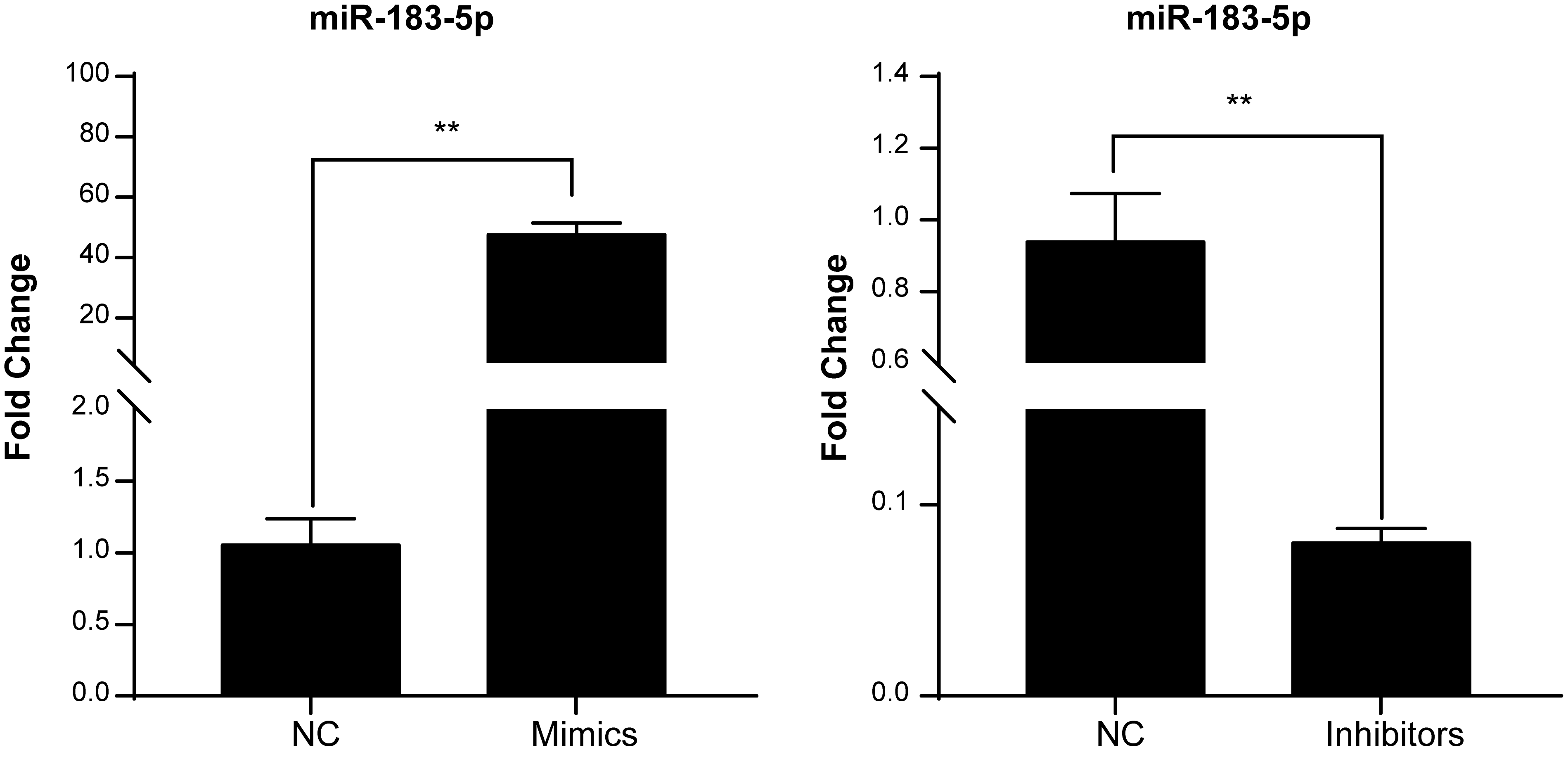

Supplement: Supplementary file 1 — Fig S1 [file JCMM-24-8614-s001.tiff]
